# *Centipeda minima* Extract Attenuates Dextran Sodium Sulfate-Induced Acute Colitis in Mice by Inhibiting Macrophage Activation and Monocyte Chemotaxis

**DOI:** 10.3389/fphar.2021.738139

**Published:** 2021-09-20

**Authors:** Brandon Dow Chan, Wing-Yan Wong, Magnolia Muk-Lan Lee, Tsz-Wing Leung, Tan-Yu Shum, William Chi-Shing Cho, Sibao Chen, William Chi-Shing Tai

**Affiliations:** ^1^ Department of Applied Biology and Chemical Technology, The Hong Kong Polytechnic University, Hung Hom, Hong Kong, SAR China; ^2^ Department of Clinical Oncology, Queen Elizabeth Hospital, Kowloon, Hong Kong, SAR China; ^3^ State Key Laboratory of Chinese Medicine and Molecular Pharmacology (Incubation), The Hong Kong Polytechnic University Shenzhen Research Institute, Shenzhen, China

**Keywords:** centipeda minima, inflammatory bowel disease, macrophages, monocytes, chemotaxis

## Abstract

Inflammatory bowel disease (IBD) is an idiopathic inflammatory disease affecting the gastrointestinal tract. IBD is characterized by courses of relapse and remission, and remains incurable. Although multiple factors are related to the pathogenesis of IBD, disruption of intestinal mucosa homeostasis has been proposed to be a major contributor to IBD, and abnormal activation of immune cells is key for initiation of the inflammatory response. Macrophages are the most abundant immune cells in the intestine. Once activated, they are responsible for secretion of pro-inflammatory cytokines and chemokines to attract circulating monocytes to inflammatory sites, exacerbating the inflammatory response, and leading to tissue damage. Therefore, the suppression of activated macrophages, cytokine/chemokine production, and subsequent monocyte chemotaxis possesses great potential for the treatment of IBD. In our study, we have demonstrated the inhibitory effect of *Centipeda minima* total extract (CME) on the activation of NF-κB, STAT3, and MAPK signaling in LPS-stimulated RAW264.7 macrophages. In addition, we identified the significant suppressive effect of CME on CCL8 expression in activated macrophages, which potentially contributed to inhibition of monocyte chemotaxis. In the DSS-induced acute colitis mouse model, we have demonstrated the suppressive effect of CME on intestinal macrophage infiltration and its ameliorative effect in IBD. Altogether, we have provided evidence of the therapeutic effect of CME in IBD and the potential of CME for the treatment of IBD.

## Introduction

Inflammatory bowel disease (IBD), comprising ulcerative colitis (UC) and Crohn’s disease (CD), is a chronic and progressive inflammatory disease affecting the gastrointestinal tract. In the past several decades, an increasing trend in the incidence of IBD has been observed worldwide, and the disease is now emergent in many countries with previously low prevalence (Cosnes et al., 2011). IBD remains incurable, and is characterized by courses of relapse and remission, with remittent abdominal pain, diarrhea, and rectal bleeding (Yu and Rodriguez, 2017). Although the exact etiology of IBD remains to be elucidated, multiple interacting factors including genetic susceptibility, environment, diet, gut microbiota, and a dysregulated immune system have been demonstrated to contribute to the pathogenesis of the disease (Ananthakrishnan, 2015). In particular, disruption of mucosal immune homeostasis in the intestine has been shown to be a major contributor to IBD (Cader and Kaser, 2013).

Intestinal homeostasis is maintained through interactions between the gut microbiota, intestinal epithelium, and host immune system. A delicate dynamic is maintained by various regulatory mechanisms, and dysregulation of any one of the three components can lead to disruption of homeostasis and an inflammatory response (Maloy and Powrie, 2011). Recently, abnormal activation of immune cells in response to the microbiota has been proposed to be central to IBD pathogenesis. Increasing evidence suggests that alteration of the host gut microbiota can trigger an immune response, and a prolonged, unresolved inflammatory response predisposes to development of IBD (Ni et al., 2017; Zuo and Ng, 2018). Macrophages belong to the innate immune system and can be found throughout the intestinal tract. As the most abundant leukocytes in the intestine, macrophages recognize pathogen-associated molecular patterns (PAMPs) on microbes and lead to activation of pro-inflammatory signaling, including the NF-κB, STAT3, and MAPK pathways. Subsequent production of pro-inflammatory cytokines, chemokines, and interferons result in recruitment of macrophages and other immune cells for the clearance of microbes (Mowat and Bain, 2011; Steinbach and Plevy, 2014; Lissner et al., 2015). However, a dysregulated response of macrophages towards commensal bacteria can lead to abnormal activation of pro-inflammatory signaling, uncontrolled production of cytokines and chemokines, and increased infiltration of immune cells, ultimately leading to tissue damage (Mowat and Bain, 2011). Therefore, suppression of dysregulated signaling pathways and cytokine/chemokine production are major focuses of IBD treatment.

Currently, various treatment options including antibiotics, corticosteroids, immunomodulators, and biologics are available for IBD patients. Although the introduction of biologics has improved the treatment of moderate-to-severe IBD patients, severe side effects such as hypersensitivity, haematological effects, autoimmune-like effects, and neurological effects hinder their use (Shivaji et al., 2019). Therefore, new therapeutics with high efficacy and minimal side effects are needed.

In recent years, increasing attention has been placed on the potential of herbal medicines for the treatment of IBD. Several clinical trials on herbal medicines for the treatment of IBD have been conducted, and their results suggest that herbal medicine possess comparable efficacy, low toxicity, and fewer side effects when compared with current IBD drugs (Ke et al., 2012; Triantafyllidi et al., 2015). Thus, herbal medicines are a vast source for a wide range of therapeutics which can be used as alternative treatments against IBD. *Centipeda minima* (L.) A. Braun and Asch. (Ebushicao) (Asteraceae) (CM) is a herbal medicine commonly used for the treatment of numerous inflammatory diseases including rhinitis, sinusitis, pain, swelling, and cancer (Chen et al., 2011; Guo et al., 2013; China, 2015; Guo et al., 2015; Mazzio et al., 2016). However, the effect of CM on IBD has not been reported. Therefore, in this study, we aimed to investigate the effect of the total extract of CM (CME) on IBD and elucidate the underlying mechanism. We found that CME exhibited potent anti-inflammatory effects against IBD, potentially through suppression of macrophage activation and monocyte chemotaxis. Our study provides evidence for CME as a promising novel alternative for the treatment of IBD.

## Materials and Methods

### Plant Materials and Sample Preparation

*Centipeda minima* was collected in Xiangfan, Hubei Province, China (latitude, 32°04′ N; longitude, 112°05′ E) and authenticated by Dr. Sibao Chen based on morphological features. A voucher specimen (EBSC-016-09) was deposited at the herbarium of the State Key Laboratory of Chinese Medicine and Molecular Pharmacology, Department of Applied Biology and Chemical Technology, The Hong Kong Polytechnic University.

Preparation of CME was conducted as described previously (Chan et al., 2016; Lee et al., 2020). In brief, CM was air-dried and ground to a coarse powder. 0.3 g CM powder was dissolved in 10 ml 50% ethanol and sonicated for 30 min at room temperature before centrifugation and collection of the supernatant. The above extraction procedure was conducted twice, and the residual powder washed once with 50% ethanol. The ethanol extracts were then combined, topped up to a final volume of 25 ml with 50% ethanol, and filtered through a 0.45 μM syringe filter before high-performance liquid chromatography (HPLC) analysis.

As previously described (Chan et al., 2016), the qualitative and quantitative analysis of chemical constituents of each batch of CME was determined using high-performance liquid chromatography coupled with quadrupole time-of-flight mass spectrometry (HPLC-QTOF-MS) and high-performance liquid chromatography diode array detection (HPLC-DAD), by comparison to an established HPLC profile with 10 chemical markers of CME.

CME was prepared in dimethyl sulfoxide (DMSO; Sigma-Aldrich, St. Louis, MO, United States) at a stock concentration of 10 mg/ml and stored at −80°C until use in *in vitro* assays. For oral administration to mice, CME was dissolved in a 0.5% carboxymethylcellulose (CMC; Sigma-Aldrich) solution.

### Cell Lines and Cell Cultures

RAW264.7 murine macrophages and THP-1 human monocytes were purchased from the American Type Culture Collection (Manassas, VA, United States). RAW264.7 and THP-1 cells were maintained in DMEM or RPMI1640 respectively, supplemented with 10% fetal bovine serum (FBS) and 50 U/ml penicillin/streptomycin (P/S) (ThermoFisher Scientific, Waltham, MA, United States) and kept at 37°C and 5% CO_2_, as described previously (Wong et al., 2016).

### Cell Viability Assay and Drug Treatment

Cell viability of RAW264.7 macrophages was examined by 3-(4,5-dimethyl-2-thiazolyl)-2,5-diphenyl-2H-tetrazolium bromide (MTT) assay (Sigma-Aldrich), performed as previously described (Wong et al., 2017).

For nitrogen oxide (NO), Western blotting, and qPCR analysis, 1 × 10^6^ RAW264.7 macrophages were incubated with 0, 0.5, 1, 2, or 4 μg/ml CME 1 h before stimulation with 1 μg/ml LPS, as indicated. Vehicle treatment served as a control. Cells and media were collected after treatment and evaluated accordingly.

### Griess Assay

NO levels from media collected from RAW264.7 macrophages were quantified by Griess Reagent System according to manufacturer’s instructions (Promega, Madison, WI, United States). Briefly, 50 μl of supernatant or standards were added to a 96-well plate, followed by 50 μl sulfanilamide solution and 50 μl N-1-napthylethylenediamine dihydrochloride (NED) solution, with a 10 min incubation period after the addition of each. Absorbance at 550 nm was measured using a Clariostar Monochromator Microplate Reader (BMG Labtech, Germany).

### Isolation of Cytoplasmic and Nuclear Fractions

Cytoplasmic and nuclear fractions in cells were isolated using NE-PER Nuclear and Cytoplasmic Extraction Reagents (Thermo Fisher Scientific) according to manufacturer’s instructions. Briefly, treated cells were collected and washed with PBS twice. Cell pellets were suspended in cytoplasmic extraction reagent I, followed by addition of cytoplasmic extraction reagent II. After centrifugation, supernatants were collected as cytoplasmic extracts. The remaining pellets were resuspended in nuclear extraction reagent and nuclear extracts were collected after centrifugation. Cytoplasmic and nuclear extracts were used for subsequent experiments.

### Western Blotting

Western blot analysis was conducted as described previously (Wong et al., 2016). Briefly, cell pellets were lysed in RIPA buffer and cell debris was removed by centrifugation. Protein concentrations were quantified using DC protein assay (Bio-Rad, Hercules, CA, United States) and were solubilized in Laemmli sample buffer. Equal protein amounts (20 µg) were loaded, electrophoresed through SDS-PAGE gels and transferred onto PVDF membranes (Bio-Rad). Blots were then blocked in 5% nonfat skim milk and probed against targeted primary antibodies and the corresponding secondary antibodies. Protein bands were visualized using Clarity Western ECL substrate (Bio-Rad) and a ChemiDoc Imaging System (Bio-Rad). Protein expression levels were quantified using Image Lab software (Bio-Rad). Antibodies used were as follows: β-actin, Histone H1, GAPDH (Santa Cruz Biotechnology, Santa Cruz, CA, United States), iNOS (BD Biosciences, CA, United States), IKK-α, IKK-β, phospho-IKK-α/β, IκB-α, phospho-IκB-α, NF-κB, phospho-NF-κB, JNK, phospho-JNK, p38, phospho-p38, STAT3, and phospho-STAT3 (Cell Signaling Technology, Danvers, MA, United States).

### Immunofluorescence Staining

Immunofluorescence staining of NF-κB was carried out as previously reported (Wong et al., 2017). Briefly, RAW264.7 macrophages were pre-treated with CME (0, 2, or 4 μg/ml) for 1 h and then stimulated with LPS (1 μg/ml) for 15 min. After fixation and permeabilization, cells were stained with antibodies against NF-κB (Cell Signaling Technology) and DAPI (ThermoFisher Scientific). Fluorescent signals were visualized using a Leica TCS SPE Confocal Microscope (Leica Microsystems, Wetzlar, Germany).

### ELISA

Concentrations of TNF-α, IL-1β, and IL-6 in cell culture media and colon cultures were measured using ELISA kits (BioLegend, Cambridge, United Kingdom) according to manufacturer’s instructions. Briefly, 96-well plates were coated with the appropriate antibodies overnight. Diluted standards or samples were added, followed by addition of detection antibodies and Avidin-HRP solution. After incubation, signal intensity was measured using a Clariostar Monochromator Microplate Reader at 450 nm, with a reference wavelength of 570 nm.

### *In vitro* Chemotaxis Assay

THP-1 chemotaxis assays were performed as described by Feige *et al.*, with slight modifications (Feige et al., 2013). Briefly, 1 × 10^6^ THP-1 cells were pre-treated with 0, 0.5, 1, 2, or 4 μg/ml CME in 100 µl 0.1% BSA/RPMI1640 for 1 h and then transferred into the upper chamber of a 24-well transwell chamber (6.5 mm diameter, 5 µm pore size, Costar, Corning, NY, United States). 600 µl of 0.1% BSA/RPMI1640 supplemented with MCP-1 (100 ng/ml) and RANTES (100 ng/ml) was added to the lower chamber and the cells were allowed to migrate for 2 h at 37°C. At the end of the experiment, cells in the lower chamber were collected and counted using a Countess II FL Automated Cell Counter (ThermoFisher Scientific).

### PCR Array and qPCR Analysis

The expression of cytokine and chemokine gene expression in RAW264.7 macrophage cell pellets were examined using a PCR array. Briefly, RAW264.7 macrophages were pre-treated with 0 or 4 μg/ml CME for 1 h and were then stimulated with 1 μg/ml LPS for 24 h. Macrophages with vehicle treatment served as a control. At the end of the experiment, cells were collected and total RNA was isolated from collected cells using the E.Z.N.A.® Total RNA Kit I (Omega Bio-tek, United States) and quantified using a Nanodrop One spectrophotometer (ThermoFisher Scientific). First strand cDNA synthesis was carried out from 0.5 μg RNA using the RT^2^ first strand kit (Qiagen, MD, United States). Diluted cDNA samples were mixed with RT^2^ SYBR Green Mastermix (Qiagen) and dispensed into each well of a 96-well RT^2^ Profiler™ PCR Array Mouse Inflammatory Response and Autoimmunity (Qiagen, Cat. # PAMM-077Z), containing primers for 90 genes related to inflammatory mediators. PCR reactions were carried out in a QuantStudio 7 Flex Real-time qPCR system (Applied Biosystems, United States), according to manufacturer’s instructions. Gene expression was analyzed using the web-based GeneGlobe Data analysis center software (Qiagen), and ≥2-fold differential expression was used as a criterion to define gene up- or down-regulation.

The expression of CCL2, CCL7, CCL8, CCL12, and CCR2 in RAW264.7 macrophages and peritoneal macrophages was determined by quantitative PCR (qPCR). Total RNA was isolated from cells and colon samples using the E.Z.N.A.® Total RNA Kit I (Omega Bio-tek), and quantified using a Nanodrop One spectrophotometer (Thermo Scientific, United States). First strand cDNA synthesis was carried out from 1 μg RNA using SuperScript® VILO™ MasterMix (ThermoFisher Scientific). PCR reaction mixtures contained 10 μl of 2X SYBR Green Master Mix (Applied Biosystems), 10 μM of forward and reverse primers, and 1 μl sample cDNA. Sequences of primers used for qPCR are listed in Table 1. Amplification was performed using the QuantStudio7 system (Applied Biosystems) at the following conditions: 2 min at 50°C, 10 min at 95°C, followed by 45 cycles of 15 sec at 95°C, and 1 min at 60°C. Relative gene expression was calculated using the 2^−∆∆CT^ method.

**TABLE 1:** Primers used in quantitative PCR analysis.

Gene	Forward primer (5′–3′)	Reverse primer (5′–3′)
CCL2	CAC​TCA​CCT​GCT​GCT​ACT​CA	GCT​TGG​TGA​CAA​AAA​CTA​CAG​C
CCL7	AAG​ATC​CCC​AAG​AGG​AAT​CTC​AA	CTT​CCC​AGG​GAC​ACC​GAC​TA
CCL8	GCT​GTG​GTT​TTC​CAG​ACC​AA	GAA​GGT​TCA​AGG​CTG​CAG​AA
CCL12	GTC​CTC​AGG​TAT​TGG​CTG​GA	CAC​TGG​CTG​CTT​GTG​ATT​CT
CCR2	TTA​CAC​CTG​TGG​CCC​TTA​TTT	CTG​AGT​AGC​AGA​TGA​CCA​TGA​C
GAPDH	TAT​GTC​GTG​GAG​TCT​ACT​GGT	GAG​TTG​TCA​TAT​TTC​TCG​T

### Animal Studies

Male and female wild-type C57BL/6 J mice were purchased from the Jackson Laboratory (Bar Harbor, ME, United States) and mated to maintain an inbred breeding colony at the Centralised Animal Facilities, The Hong Kong Polytechnic University. All mice were kept in a barrier-sustained animal house, air-conditioned at 20 ± 2°C, and humidity at 55 ± 10%, under a 12 h light/dark cycle. Food and water were available *ad libitum*. All animal experiments were approved by the Animal Subjects Ethics Sub-Committee (ASESC) of The Hong Kong Polytechnic University and conducted in accordance with the Institutional Guidelines and Animal Ordinance of the Department of Health, HK S.A.R.

### *In vivo* Peritoneal Monocyte Chemotaxis

The *in vivo* peritoneal monocyte chemotaxis experiment was conducted as described by Feige et al. (2013). Briefly, female C57BL/6J mice were randomly assigned to 3 groups (*n* = 7 per group) and were treated by daily oral gavage as follows: 1) 0.5% CMC (vehicle); 2) 250 mg/kg CME in 0.5% CMC (CME-LD); and 3) 500 mg/kg CME in 0.5% CMC (CME-HD), for 8 consecutive days. At day 5, mice received 3% thioglycollate intraperitoneal (i.p.) injections to induce monocyte chemotaxis. At day 8, mice were sacrificed and cells were collected from the peritoneal cavity. Cells were washed with ice-cold PBS 3 times and counted using a Countess II FL Automated Cell Counter (ThermoFisher Scientific). The remaining cells were stored at −80°C until use.

### DSS-Induced Acute Colitis and Assessment

The acute colitis animal study was performed as described previously, with minor modifications (Wong et al., 2016). Briefly, 8-week-old male wild type C57BL/6J mice were randomly assigned into 3 groups (*n* = 12 per group) and acute colitis was induced in mice by providing 2.5% w/v DSS (reagent grade; 36,000–50,000 Da; MP Biomedicals, Solon, OH, United States) in drinking water. Mice were treated with vehicle, CME-LD, or CME-HD by oral gavage, daily for 7 consecutive days. An uninduced control group (*n* = 11) treated with vehicle was also included. Body weight, and food and water consumption were recorded daily. Disease activity index (DAI) was determined as described previously (Wong et al., 2017). Mice were sacrificed at day 7, intestines were removed, and colon lengths were measured. Colons were then washed with PBS, fixed in formalin solution, and histological analysis performed using H&E and immunohistochemical staining as previously described (Wong et al., 2016; Wong et al., 2017).

### Colon Tissue Culture

Colon tissue culture was conducted as described previously (Wong et al., 2017). Briefly, 1 cm colon sections were removed from mice during sacrifice and washed thoroughly with PBS. Colon sections were then incubated in RPMI1640 medium (ThermoFisher Scientific) for 24 h. Conditioned media was then collected and cytokine levels were quantified using ELISA.

### Statistical Analysis

Statistical analyses of the differences between two groups of interest were performed using the unpaired Student’s *t*-test. Statistical significance was indicated as *p < 0.05, **p < 0.01, and ***p < 0.001. Data are presented either as mean ± SD of three independent experiments (*in vitro* experiments) or mean ± SEM (*in vivo* experiments).

## Results

### CME Suppressed NO Production and iNOS Expression in LPS-Stimulated RAW264.7 Macrophages

**FIGURE 1:**
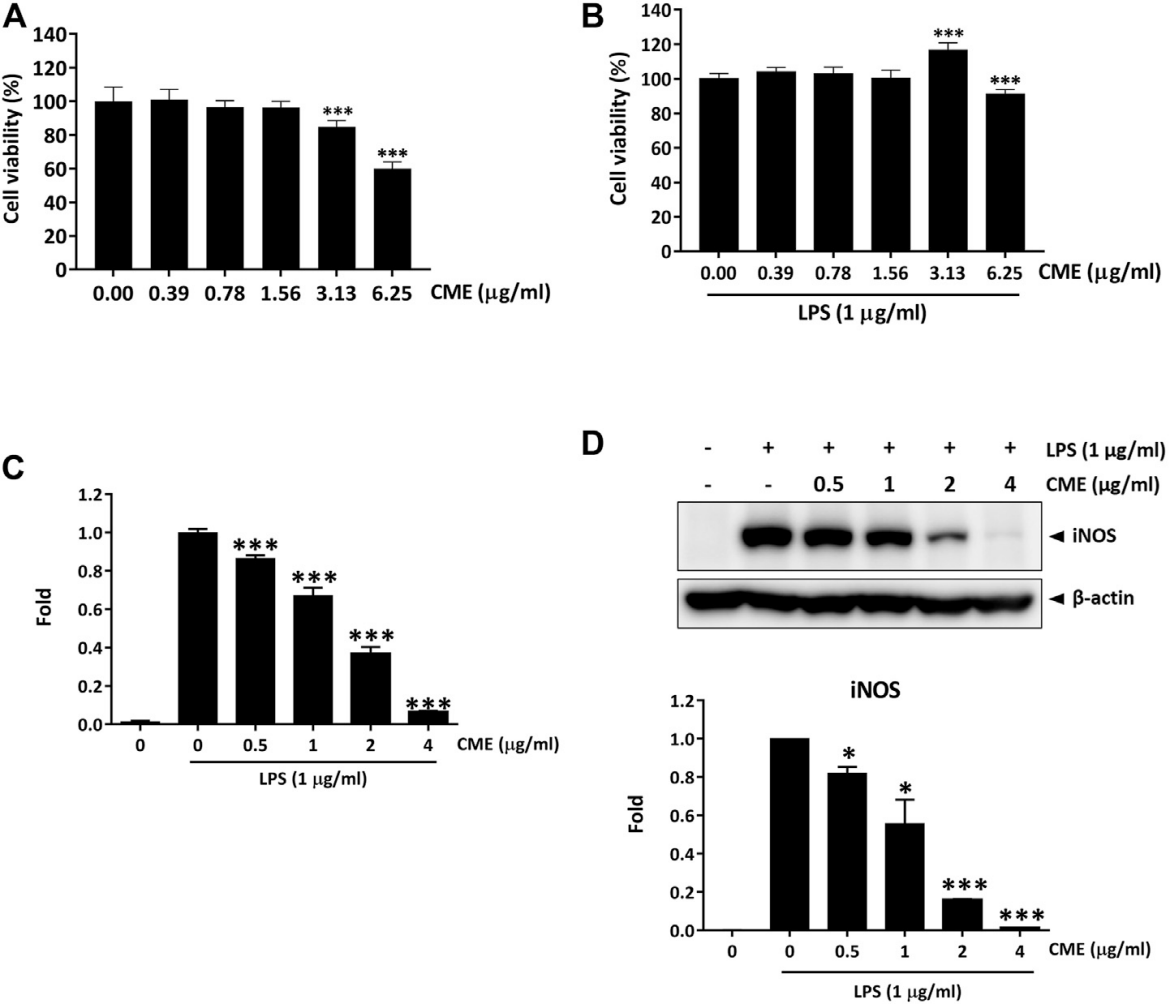
CME suppressed the production of NO and expression of iNOS in LPS-stimulated RAW264.7 macrophages. Cell viability of RAW264.7 macrophages after **(A)** 24 h treatment with CME or **(B)** 24 h co-treatment with CME and LPS (1 μg/ml). **(C)** NO production levels and **(D)** iNOS expression levels of LPS-stimulated RAW264.7 macrophages after 24 h treatment with CME (0–4 μg/ml). Data are expressed as mean ± SD; Unpaired *t*-test, *p < 0.05, **p < 0.01, ***p < 0.001 compared to LPS-stimulated control.

Previous studies have demonstrated the anti-inflammatory effect of the aqueous extract of *Centipeda minima* in λ-carrageenan-induced paw edema in mice (Huang et al., 2013), however, the potential effect of CME in IBD remains to be identified. To evaluate the effect of CME and identify its potential mechanisms of action, we first examined the effect of CME on macrophages, key players in the pathogenesis of IBD (Steinbach and Plevy, 2014). In order to determine optimal doses for downstream experiments, we tested the cell viability of RAW264.7 murine macrophages after CME treatment. As shown in Figures 1A,B, CME exhibited similar cytotoxicity in unstimulated and LPS-stimulated RAW264.7 murine macrophages at the tested concentrations (0, 0.39, 0.78, 1.56, 3.13, and 6.25 μg/ml). Based on these results, non-toxic doses of 0.5, 1, 2, and 4 μg/ml CME were selected for downstream experiments.

Although production of NO is part of the antibacterial response of the intestine upon infection, uncontrolled NO production is harmful (Kolios et al., 2004). Therefore, suppression of substantial NO production and the expression of iNOS, the enzyme responsible for NO production, is important for the treatment of IBD. Figures 1C,D show that CME dose-dependently suppressed the production of NO and expression of iNOS, suggesting that CME possessed potential anti-inflammatory effects.

### CME Mitigates NF-κB and STAT3 Signaling in LPS-Stimulated RAW264.7 Macrophages

**FIGURE 2:**
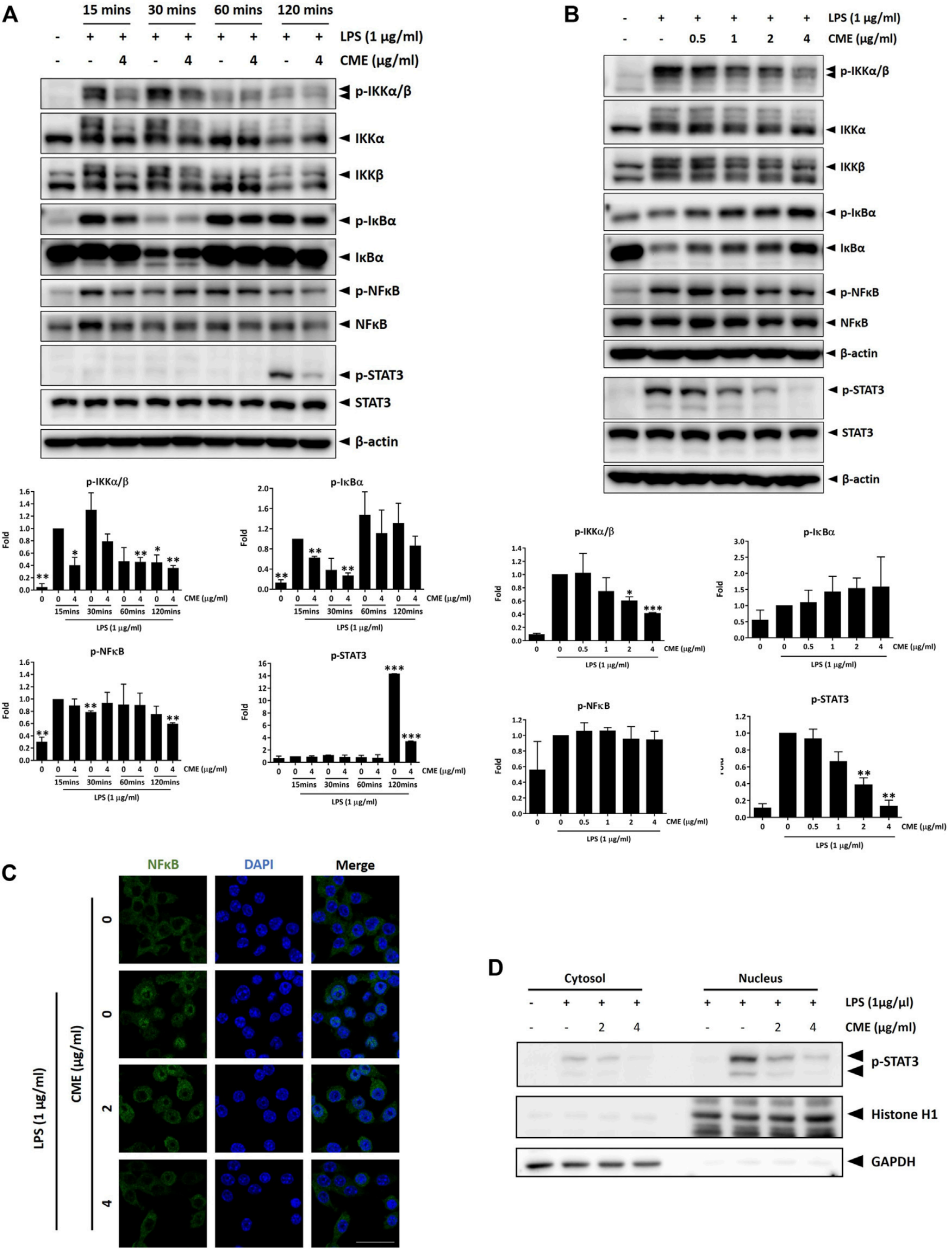
CME suppressed NF-κB and STAT3 signaling in LPS-stimulated RAW264.7 macrophages. Cells were pre-treated with CME at 4 μg/ml for 1 h and then stimulated with LPS for the indicated times in time-dependent experiments, or with CME (0–4 μg/ml) for 1 h and then stimulated with LPS (1 μg/ml) for 15 min (NF-κB signaling)/120 min (STAT3 signaling) for dose-dependent experiments. Lysates were probed with the indicated antibodies and representative Western blot images of **(A)** time-dependent and **(B)** dose-dependent experiments are shown. Quantification of phosphorylated IKKα/β, IκBα, NF-κB, and STAT3 protein expression are displayed under the corresponding blot images. Density values were compared to the LPS-stimulated control. **(C)** Cells were pre-treated with CME (0–4 μg/ml) for 1 h and then stimulated with LPS (1 μg/ml) for 15 min. Cells were stained for NF-κB (green) and nuclei stained with DAPI (blue). **(D)** Cells were pre-treated with CME (0, 2, and 4 μg/ml) for 1 h and then stimulated with LPS (1 μg/ml) for 2 h. Nuclear and cytoplasmic fractions were isolated and phosphorylated STAT3 detected by Western blot. Histone H1 and GAPDH were used as loading controls for the nuclear and cytoplasmic fractions respectively. Data are expressed as mean ± SD; Unpaired *t*-test, *p < 0.05, **p < 0.01, and ***p < 0.001 compared to LPS-stimulated control. Scale bar, 25 µm.

Dysregulation of the NF-κB signaling pathway has been associated with initiation and progression of IBD, and excessive production of pro-inflammatory cytokines due to abnormal activation of NF-κB signaling has been correlated with inflammation in the intestine (Han et al., 2017). Further, emerging evidence has also demonstrated that STAT3 signaling contributes to the pathogenesis of IBD (Sugimoto, 2008), emphasizing the importance of both NF-κB and STAT3 signaling in IBD. To examine the effect of CME on the expression of signaling molecules in the aforementioned signaling pathways, we performed a time-dependent experiment in RAW264.7 macrophages. Cells pre-treated with vehicle or 4 μg/ml CME were stimulated with LPS for 15, 30, 60, and 120 min. As shown in Figure 2A, CME suppressed phosphorylation of IKKα/β, IκBα, NF-κB, and STAT3 in LPS-stimulated RAW264.7 macrophages in a time-dependent manner, suggesting the inhibitory effect of CME on the activation of NF-κB and STAT3 signaling in LPS-stimulated macrophages.

To further evaluate the anti-inflammatory effect of CME, we examined the dose-dependent effect of CME in LPS-stimulated RAW264.7 macrophages. Based on our time-dependent experiments, the 15 min timepoint was selected for this experiment. As the expression of phosphorylated STAT3 was seen to be upregulated only after 120 min of stimulation with LPS, the 120 min timepoint was used for STAT3. In line with our time-dependent experiments, CME dose-dependently suppressed the expression of phosphorylated IKKα/β, IκBα, NF-κB, and STAT3 in LPS-stimulated RAW264.7 macrophages (Figure 2B).

NF-κB translocates into the nucleus upon activation, triggering the transcription of pro-inflammatory genes and activating the inflammatory cascade. Therefore, suppression of NF-κB translocation can induce inhibition of the inflammatory response. As shown in Figure 2C, LPS induced the translocation of NF-κB from the cytoplasm to the nucleus, resulting in colocalization of NF-κB with the nucleus. Treatment with CME dose-dependently suppressed nuclear translocation of NF-κB, suggesting inhibition of NF-κB signaling by CME. Like NF-κB, upon activation STAT3 also translates into the nucleus to trigger downstream activation of pro-inflammatory signaling. To confirm our above observation on the inhibition of STAT3 signaling by CME, we further investigated the subcellular expression of phosphorylated STAT3. In line with our results above, CME treatment dose-dependently decreased the amount of activated STAT3 in the nuclear fraction (Figure 2D), supporting its inhibitory activity on this signaling pathway.

Altogether, in LPS-stimulated RAW264.7 macrophages, CME suppressed the activation of NF-κB and STAT3 signaling, major signaling pathways in the pathogenesis of IBD.

**FIGURE 3:**
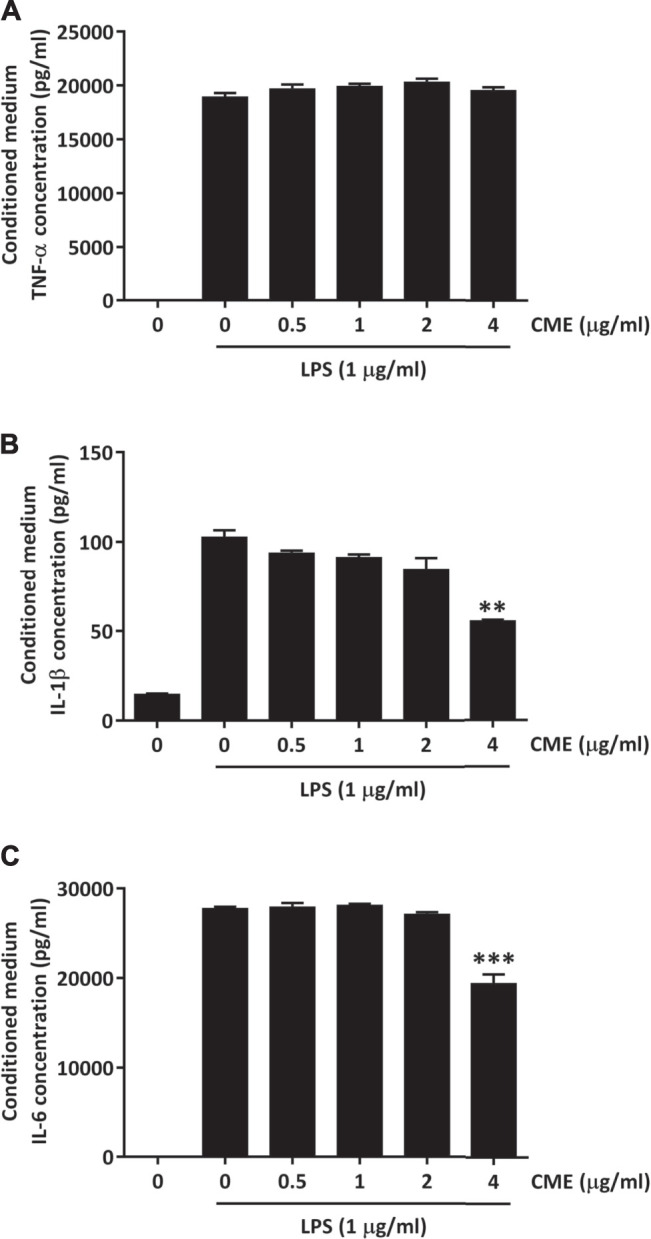
CME suppressed pro-inflammatory cytokine production in LPS-stimulated RAW264.7 macrophages. Cells were treated with CME (0–4 μg/ml) and stimulated with LPS (1 μg/ml) for 24 h. Media were collected and the concentrations of **(A)** TNF-α, **(B)** IL-1β, and **(C)** IL-6 were quantified using ELISA. Data are expressed as mean ± SD; Unpaired *t*-test, **p < 0.01 and ***p < 0.001 compared to LPS-stimulated control.

As activation of NF-κB and STAT3 signaling can lead to excessive production of pro-inflammatory cytokines, we next evaluated the effect of CME on TNF-α, IL-1β, and IL-6 production in LPS-stimulated RAW264.7 macrophages. Although CME treatment did not suppress the production of TNF-α, we observed significant reductions in IL-1β and IL-6 production (Figures 3A–C). When LPS-stimulated macrophages were treated with 4 μg/ml CME, the production of IL-1β and IL-6 was suppressed by 45 and 30%, respectively. Our results demonstrated that CME suppressed both NF-κB and STAT3 signaling, resulting in inhibition of IL-1β and IL-6 production.

**FIGURE 4:**
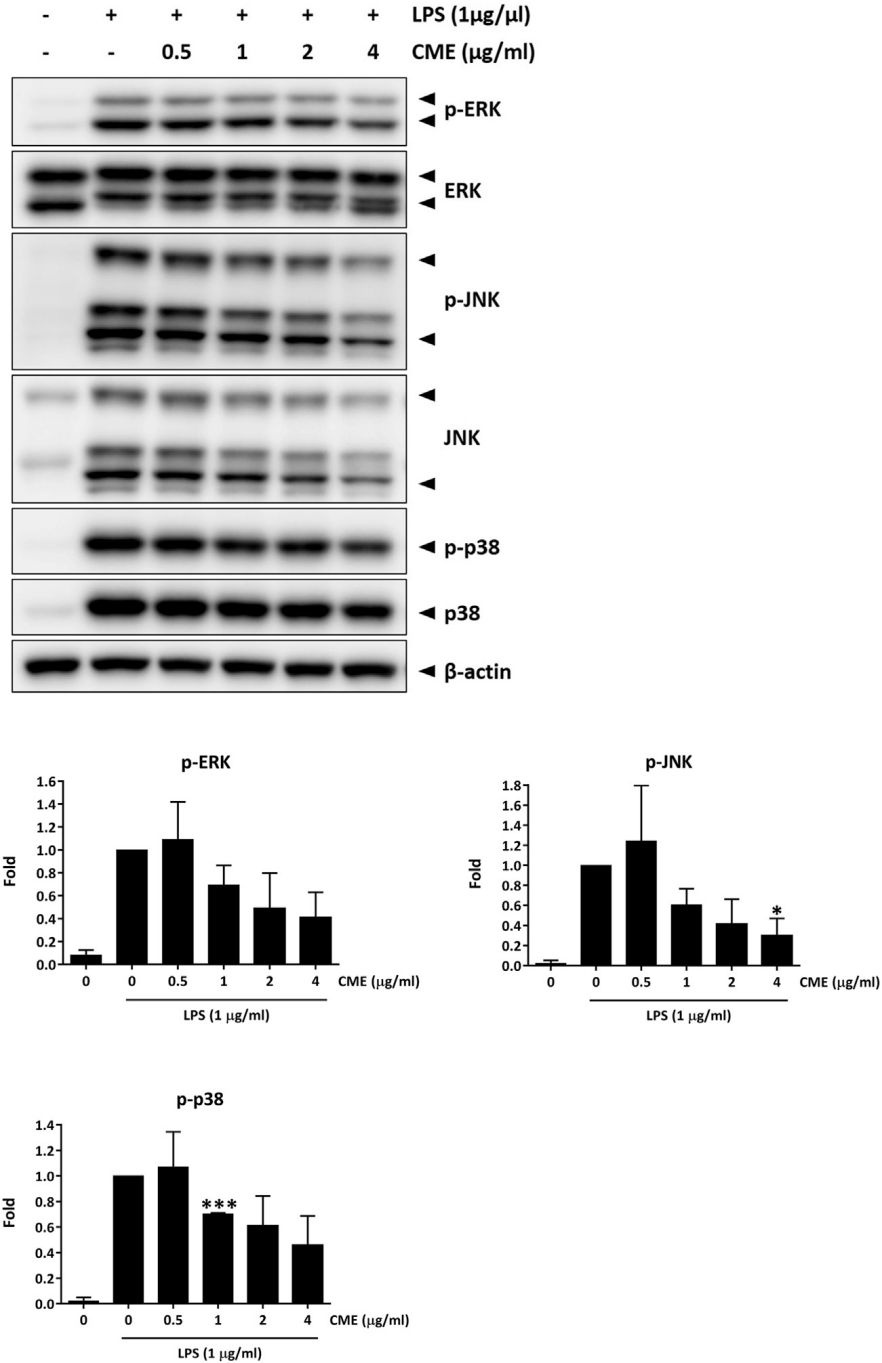
CME suppressed MAPK signaling in LPS-stimulated RAW264.7 macrophages. Cells were pre-treated with CME (0–4 μg/ml) for 1 h and then stimulated with LPS (1 μg/ml) for 15 min. Lysates were probed with the indicated antibodies. Representative Western blot images and quantification of phosphorylated ERK, JNK and p38 protein expression are shown. Data are expressed as mean ± SD; Unpaired *t*-test, *p < 0.05 compared to LPS-stimulated control.

To elucidate the downstream effect of CME, we also examined the expression of the MAPKs, the downstream effectors of NF-κB. As shown in Figure 4, at doses of 1 μg/ml or higher, CME dose-dependently suppressed the expression of phosphorylated ERK, JNK, and p38 in LPS-stimulated RAW264.7 macrophages. After treatment with 4 μg/ml CME, the expression of phosphorylated ERK, JNK, and p38 were suppressed by 59, 70, and 54%, respectively, suggesting CME also inhibited MAPK signaling.

### CME Suppressed *in vitro* and *in vivo* Expression of Pro-Inflammatory Cytokines and Chemokines and Mitigated Monocyte Chemotaxis

**FIGURE 5:**
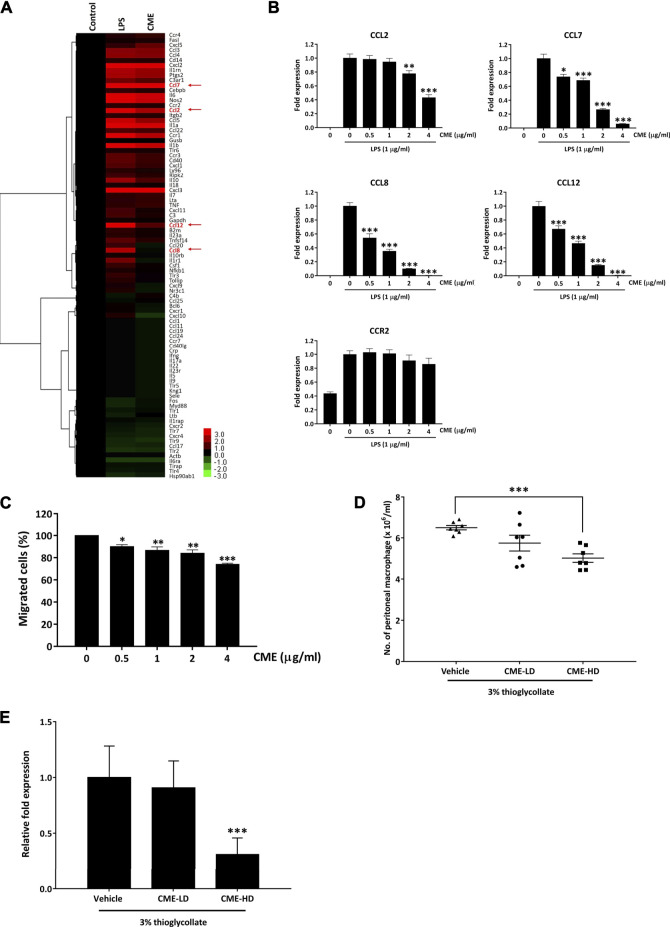
CME suppressed pro-inflammatory cytokine and chemokine expression in LPS-stimulated RAW264.7 macrophages and inhibited monocyte chemotaxis. **(A)** RAW264.7 macrophages were pre-treated with CME (0 or 4 μg/ml) for 1 h and then stimulated with LPS (1 μg/ml) for 24 h. Untreated and uninduced cells were used as controls. RNA was isolated from cells and PCR array analysis was performed. Hierarchical clustering was performed to show the differentially expressed genes in the samples. Statistically significant genes selected for further validation are highlighted in red. **(B)** RAW264.7 macrophages were pre-treated with CME (0–4 μg/ml) for 1 h and then stimulated with LPS (1 μg/ml) for 24 h. RNA was isolated from cells and gene expression levels of CCL2, CCL7, CCL8, CCL12, and CCR2 were assessed by qPCR. **(C)** THP-1 monocytes were pre-treated with CME (0–4 μg/ml) for 1 h and then allowed to migrate towards MCP-1 (100 ng/ml) and RANTES (100 ng/ml) for 2 h. Migrated cells were counted and normalized with control. **(D)** Female C57BL/6 J mice were treated with vehicle (0.5% CMC), CME-LD (250 mg/kg in 0.5% CMC), or CME-HD (500 mg/kg in 0.5% CMC) for 8 days. 3% thiogallate was i.p. injected into the mice at day 5, migrated macrophages were collected from the peritonea at the time of sacrifice, and cell numbers were counted (*n* = 7 per group). **(E)** RNA was isolated from peritoneal macrophages collected from mice and the gene expression level of CCL8 was analyzed. The CCL8 levels were normalized to vehicle treatment. Data are expressed as mean ± SD for gene expression analysis in RAW264.7 macrophages and THP-1 monocytes, and mean ± SEM for peritoneal macrophages; Unpaired *t*-test, *p < 0.05, **p < 0.01, and *p < 0.001 compared to LPS-stimulated control or vehicle control.

It has been shown that in IBD, increased production of cytokines or chemokines by an overreaction of macrophages to gut microbiota in the intestine leads to increased immune cell infiltration and subsequent tissue damage (Cader and Kaser, 2013). To identify if CME could alter cytokine or chemokine signaling, we performed PCR array analysis to examine the mRNA expression of cytokines and chemokines in LPS-stimulated RAW264.7 macrophages after treatment with CME. Among the differentially expressed genes, CME significantly suppressed the expression of monocyte chemokine attractants CCL2, CCL7, CCL8, and CCL12 in macrophages when compared to vehicle treatment (Figure 5A), suggesting the potential inhibitory effect of CME on monocyte chemotaxis. In particular, we saw significant downregulation of CCL8 expression in LPS-stimulated macrophages treated with CME. In comparison with vehicle-treated LPS-stimulated macrophages, the expression of CCL8 in CME-treated macrophages was much reduced (0.00364 fold). As it is known that these ligands attract monocytes expressing the chemokine receptor CCR2, we also examined whether CME treatment could suppress the expression of CCR2. However, according to results from the PCR array, the expression of CCR2 was only mildly downregulated after CME treatment (0.741 fold), suggesting that CME potentially suppressed monocyte chemotaxis via chemokine production rather than receptor expression.

Based on the above PCR array results, we further validated the suppressive effect of CME on CCL2, CCL7, CCL8, CCL12, and CCR2 in LPS-stimulated macrophages via qPCR. In accordance with our PCR array results, CME significantly suppressed the expression of CCL2, CCL7, CCL8, and CCL12 in LPS-stimulated macrophages, in a dose-dependent manner (Figure 5B). Although we also observed a slight suppressive effect of CME on CCR2, this effect was not comparable to the inhibitory effect on the aforementioned chemokines (Figure 5B). Therefore, we postulated that CME potentially suppressed monocyte chemotaxis to the site of inflammation site via inhibition of monocyte chemoattractant production.

To confirm the effect of CME on monocyte chemotaxis, we employed an *in vitro* chemotaxis assay. As shown in Figure 5C, CME suppressed the migration of human monocyte THP-1 cells towards MCP-1 and RANTES in a dose-dependent manner. Treatment with 4 μg/ml CME significantly inhibited the migration of THP-1 cells by approximately 27% when compared to vehicle treatment. In addition, we also utilized a thioglycollate-induced peritonitis mouse model to examine the *in vivo* inhibitory effect of CME on monocyte chemotaxis (Feige et al., 2013). In this model, induction of a “sterile” inflammatory response in the peritoneum through injection of thioglycollate results in recruitment of peripheral monocytes which later differentiate into macrophages (Li et al., 1997). Mice were treated with vehicle, 250 mg/kg (CME-LD), or 500 mg/kg (CME-HD) and then induced with thioglycollate. Monocytes attracted to the peritoneal cavity were collected and counted after mice were sacrificed. Compared to mice treated with vehicle, CME exhibited a dose-dependent suppressive effect on macrophage accumulation in the peritoneal cavity; a 12 and 23% reduction in the number of peritoneal macrophages was observed in CME-LD and CME-HD treated mice respectively (Figure 5D). Furthermore, to validate the inhibitory effect of CME *in vivo*, as we identified a significant reduction in CCL8 expression in *in vitro* experiments, we examined CCL8 levels in peritoneal macrophages from thioglycollate-induced mice. As shown in Figure 5E dose-dependent inhibition of CCL8 was observed in peritoneal macrophages isolated from CME treated mice, with a 9 and 69% suppression of CCL8 expression in CME-LD and CME-HD treated mice respectively, compared to control. In summary, these data suggest that CME inhibited monocyte chemotaxis through suppression of monocyte chemoattractants, and potentially via CCL8.

### CME Attenuated DSS-Induced Acute Colitis in Mice

**FIGURE 6:**
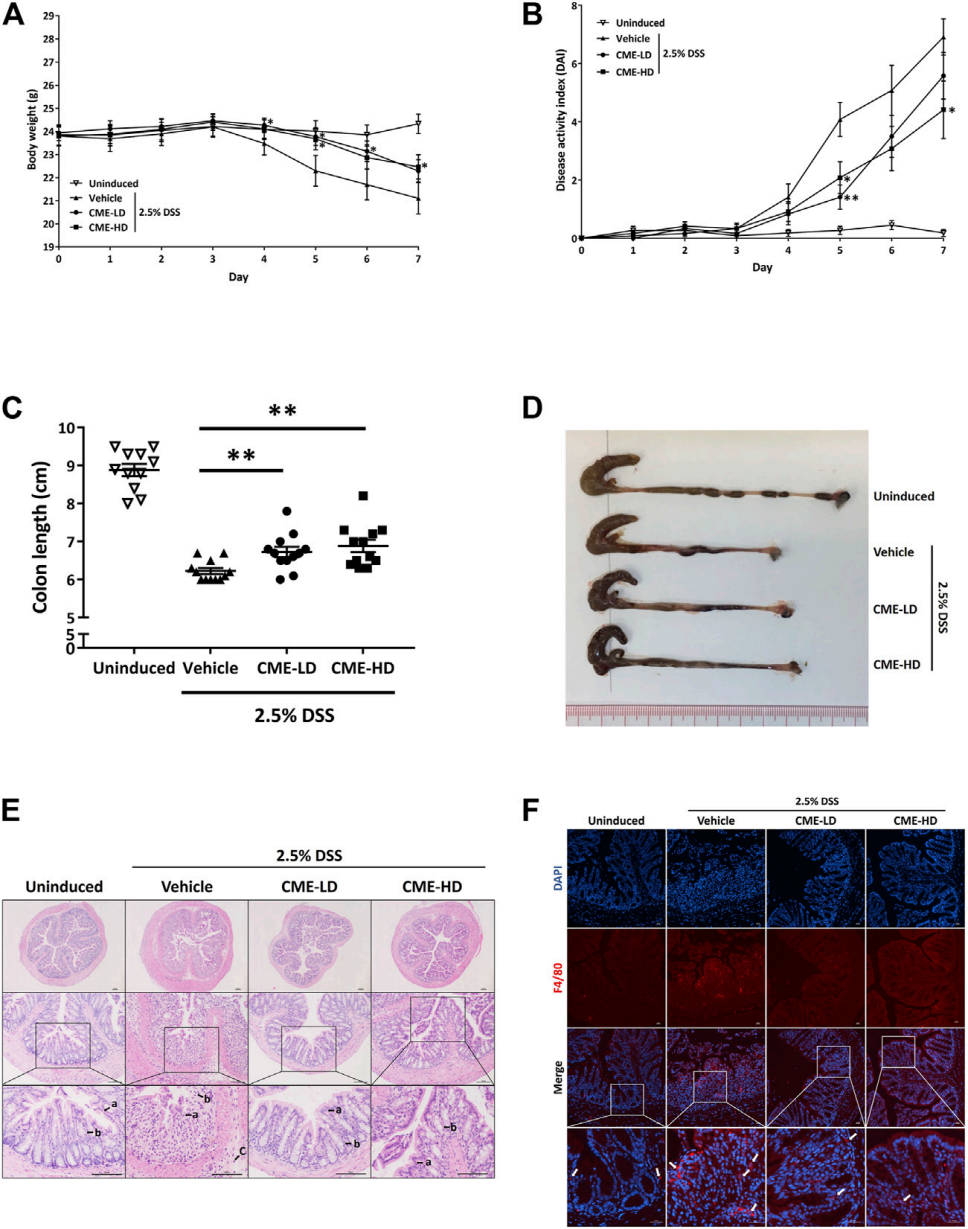
CME attenuated DSS-induced acute colitis in mice. Male C57BL/6 J mice were administered 2.5% DSS in their drinking water for 7 days to induce acute colitis. Mice were orally fed with vehicle, CME-LD or CME-HD during the induction time (*n* = 12 per group). An uninduced group fed with vehicle only served as a control (*n* = 11 per group). **(A)** Body weight changes and **(B)** DAI were recorded daily. At day 7, mice were sacrificed, and **(C)** colon lengths were measured. **(D)** Representative image of colon lengths of mice. **(E)** H&E histological analysis of colon sections. a) epithelial lining; b) colon crypts; and c) infiltration of immune cells. Scale bar, 100 µm. **(F)** Colon sections were stained using F4/80 (red) and DAPI (blue). White arrows indicate macrophages. Scale bar, 25 µm. Data are expressed as mean ± SEM; Unpaired *t*-test, *p < 0.05, **p < 0.01, and ***p < 0.001 compared to DSS-induced control.

To study the *in vivo* effect of CME in IBD, we employed the DSS-induced acute colitis mouse model, a commonly used animal model for the study of IBD. With DSS, the body weight of mice decreased gradually from day 4 post-induction, while CME-LD and CME-HD treatments ameliorated this weight loss (Figure 6A). The overall condition of the mice was evaluated via assessment of disease activity index (DAI), a combined score of body weight, stool consistency, and fecal blood assessments. As shown in Figure 6B, DAI scores were increased significantly in vehicle-treated mice after 4 days of DSS induction, indicating the disease progression caused by DSS. In comparison, DAI scores were decreased in a dose-dependent manner in mice treated with CME, suggesting that CME could improve the condition of DSS-induced mice.

**FIGURE 7:**
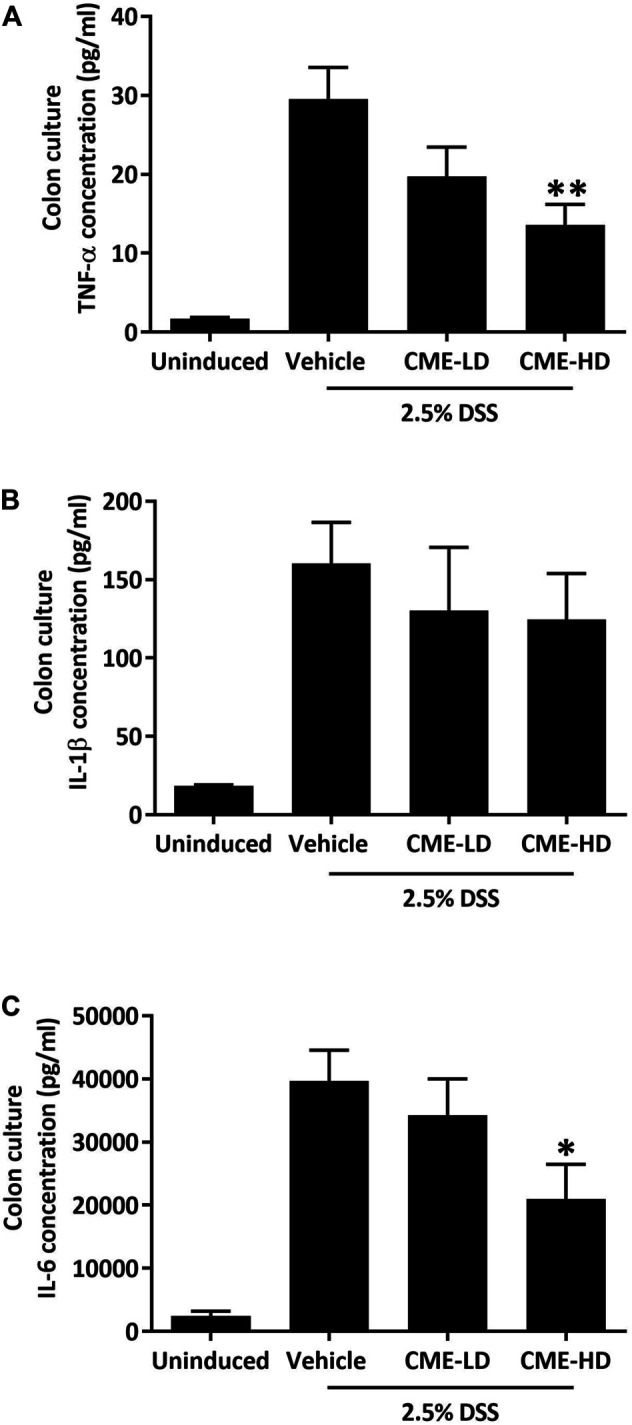
CME suppressed pro-inflammatory cytokine production in colons of DSS-induced mice. After sacrifice, colon sections were removed from mice and cultured in RPMI1640 media for 24 h. Media were collected and the concentrations of **(A)** TNF-α, **(B)** IL-1β, and **(C)** IL-6 were quantified using ELISA. Data are expressed as mean ± SEM; Unpaired *t*-test, *p < 0.05 and **p < 0.01 compared to DSS-induced control.

As colon shortening is a key feature of DSS-induced colitis mice, we examined the colon lengths of colitis mice treated with vehicle, CME-LD, or CME-HD. As shown in Figures 6C,D, average colon length was decreased by approximately 30% in DSS-induced mice when compared to uninduced mice. After CME treatment, a dose-dependent increase in colon length was observed. Colon lengths of mice treated with CME-LD and CME-HD were significantly increased by 8 and 11%, respectively. H&E staining of mouse colon tissues also indicated the therapeutic efficacy of CME. In colons of DSS-induced mice, typical colitis morphology, including destruction of epithelial cell architecture, loss of crypts, and infiltration of inflammatory cells was observed (Figure 6E). CME treatment ameliorated the damage induced by DSS—epithelial cell architecture and crypts were preserved, and there was a reduction in inflammatory cell infiltration. To validate the inhibitory effect of CME on macrophage chemotaxis, we also examined the degree of macrophage infiltration in mice colons, via immunofluorescent staining. As shown in Figure 6F, in DSS-induced mice, increased macrophage infiltration in colons was observed, while CME treatment suppressed infiltrating macrophage counts. Further, we examined the production of pro-inflammatory cytokines from mice colons to evaluate the effects of inhibition of macrophage chemotaxis. Upon induction of colitis, significant increases in TNF-α, IL-1β, and IL-6 production were observed in mouse colons, and CME treatment could dose-dependently reduce the production of these cytokines (Figures 7A–C). The levels of TNF-α, IL-1β, and IL-6 in mice treated with CME-HD were suppressed by 54, 22, and 47%, respectively.

## Discussion

Monocytes and macrophages are key components of the innate immune system and participate in the maintenance of intestinal immune homeostasis. Upon tissue damage, innate immune cells recognize damage-associated molecular patterns (DAMPs) and pathogen-associated molecular patterns (PAMPs) and attract neutrophil infiltration. Activated neutrophils recruit monocytes, which are responsible for direct removal of pathogens and damaged tissues, to the site of inflammation and lead to the resolution of inflammation (Na et al., 2019). However, in IBD, inappropriate recognition of the gut microbiota by monocytes and macrophages leads to substantial activation of the immune system and disruption of intestinal homeostasis. A major feature of IBD is an abnormal activation of cytokines and chemokines. These pro-inflammatory mediators secreted by activated immune cells can recruit additional immune cells from the circulation into the inflamed tissue, exacerbating the inflammatory response. Chronic, relapsing activation of the immune system can lead to unresolved inflammation, substantial damage of the epithelial barrier, and intestinal injury (Maloy and Powrie, 2011; Ni et al., 2017; Zuo and Ng, 2018). Therefore, suppression of aberrantly activated monocytes and macrophages and subsequent production of inflammatory mediators are of increasing interest in the treatment of IBD (Na et al., 2019).

Currently, immunosuppressive agents, biologics, and small molecules are employed for the treatment of IBD. Many of these agents target the receptors responsible for activation of the inflammatory response, or directly inhibit pro-inflammatory transcription factors such as NF-κB and JAK. These inhibitory actions result in the suppression of downstream effectors and finally, suppression of the inflammatory response. Although promising effects have been observed in IBD patients, high doses, development of drug-dependency, and serious side effects including hypersensitivity and immunosuppression have been observed (Bantel et al., 2000; Mowat et al., 2011; Oakley and Cidlowski, 2013; Probert, 2013; Rawla et al., 2018; Sabino et al., 2019). As a result, effective and safe therapeutics for the treatment of IBD are in demand.

Herbal medicines have been used in Asian countries for the treatment of diseases for centuries, and a continually increasing trend in the use of herbal remedies has been observed among IBD patients (Comar and Kirby, 2005; Tillisch, 2007). Numerous clinical trials using herbal formulas or single herbs for the treatment of IBD have shown superior effects compared to commonly used IBD therapeutics, suggesting the potential of herbal medicines for the treatment of IBD (Ke et al., 2012).

Here we have conducted a systematic study to elucidate the anti-inflammatory effect of the herbal extract, CME, and its potential therapeutic effect in IBD. We first employed RAW264.7 murine macrophages to examine the effect of CME on NF-κB signaling, one of the central signaling pathways in innate immunity (Liu et al., 2017). We identified significant and time-dependent suppression of IKK and IκBα phosphorylation after treatment with CME in LPS-stimulated RAW264.7 macrophages, suggesting that the inhibitory effect of CME is exhibited upstream of NF-κB. Further dose-dependent experiments also demonstrated the inhibitory effect of CME on IKK phosphorylation, and although we did not observe similar effects on IκBα, we postulate that there may be delayed effects on its inhibition. More importantly, we demonstrated there was a dose-dependent suppression of the nuclear translocation of NF-κB and pro-inflammatory cytokine production after CME treatment, suggesting the potent inhibitory effect of CME on NF-κB signaling.

The MAPK signaling pathways have been implicated in the pathogenesis of IBD, potentially via modulation of the levels of pro-inflammatory cytokines. Increased expression of phosphorylated ERK, JNK, and p38 have been observed in the intestinal mucosa of active IBD patients (Waetzig et al., 2002), and treatment with MAPK inhibitors could ameliorate the production of pro-inflammatory cytokines in animal models and isolated colonic tissues (Waetzig et al., 2002; Hollenbach et al., 2004; Assi et al., 2006). The therapeutic effect of MAPK inhibitors in IBD has also been studied in clinical trials, where inhibition of MAPK resulted in clinical improvement and remission (Hommes et al., 2002; Dotan et al., 2010). Therefore, MAPK inhibitors harbor great potential for the treatment of IBD. In our study, we have demonstrated that in addition to NF-κB signaling, CME also inhibited the activation of MAPK signaling members p38, ERK, and JNK, suggesting the multi-targeting effect of CME for treatment of inflammation.

Monocyte chemotaxis plays an important role in mediating the inflammatory process. However, aberrant recruitment of monocytes and subsequent secretion of pro-inflammatory cytokines can lead to substantial tissue damage at the site of injury. Increased infiltration of dysregulated macrophages derived from circulating monocytes into the intestinal mucosa and increased levels of pro-inflammatory cytokines such as IL-1, IL-6, and TNF-α have been observed in patients with active IBD (Mahida, 2000; Wang et al., 2009). Additionally, the level of chemokine elevation is strongly correlated with disease activity in IBD patients (Gijsbers et al., 2006). Therefore, inhibition of chemotaxis to sites of inflammation is an attractive approach to suppress uncontrolled inflammatory signals. In our study, we demonstrated that CME could suppress levels of various cytokines and chemokines, and in particular, we identified substantial suppression of CCL8 expression in LPS-stimulated macrophages and peritoneal macrophages after CME treatment. CCL8 is a pro-inflammatory chemokine that plays an important role in mediating the inflammatory response through recruitment of inflammatory monocytes (Asano et al., 2015). It has been shown that CCL8 was overexpressed in the intestines of DSS-induced mice and active IBD patients, and inhibition of CCL8 exhibited a potential therapeutic effect in DSS-induced mice (Asano et al., 2015; Jones et al., 2018). Therefore, CME may exhibit anti-inflammatory effects via suppression of CCL8 and the subsequent inhibition of inflammatory monocyte chemotaxis. We demonstrated the strong inhibitory effects of CME on monocyte chemotaxis *in vitro* and also in the intestines of DSS-induced mice, suggesting the therapeutic effect of CME in IBD may occur through inhibition of chemotaxis.

In conclusion, macrophages have emerged as therapeutic targets in IBD, and treatments focused on suppression of aberrantly activated macrophages, secretion of pro-inflammatory cytokines and chemokines, and monocyte chemotaxis have been studied. Our study has demonstrated the significant inhibitory effect of CME on LPS-stimulated RAW264.7 murine macrophages and the expression of pro-inflammatory cytokines and chemokines. We have also shown the strong suppressive effect of CME on chemotaxis and its therapeutic effect in DSS-induced acute colitis mice, suggesting the potential of CME for the treatment of IBD.

## Data Availability

The original contributions presented in the study are included in the article/Supplementary Material, further inquiries can be directed to the corresponding author.
